# Editorial: Site specific imaging guidelines in head & neck, and skull base cancers

**DOI:** 10.3389/fonc.2024.1357215

**Published:** 2024-01-18

**Authors:** Richa Vaish, Abhishek Mahajan, Sarbani Ghosh Laskar, Kumar Prabhash, Vanita Noronha, Anil K. D’Cruz

**Affiliations:** ^1^ Tata Memorial Hospital, Homi Bhabha National Institute, Mumbai, India; ^2^ Radiology, The Clatterbridge Cancer Centre NHS Foundation Trust, Liverpool, United Kingdom; ^3^ Oncology-Apollo Group of Hospitals, Department of Oncology, Apollo Hospital, Navi Mumbai, India

**Keywords:** head and neck neoplasm, imaging guidelines, skull base neoplasm, NIRADS, TIRADS, artificial intelligence-AI

## Introduction

Imaging is an integral component in the management plan for any cancer, which is multifaceted. It helps with various aspects, including diagnosis, treatment planning, treatment response assessment, and follow-up (1–8). There has been a boom in the field of imaging with new and sophisticated imaging machines. While this gamut of imaging tools is essential and improves the quality and information provided by the images, it is equally important to standardize the reporting methods and lay down imaging guidelines to convey this information accurately to treating clinicians (9, 10). There may be a lack of direct interaction between radiologists and clinicians, which is commonplace due to the increasing role of remote viewing stations (11). Traditional reporting was more in the form of free text and narratives. This could obscure the information in the long narratives, making interpretation difficult for clinicians and compromising clarity. Therefore, structured reporting has been increasingly emphasized to circumvent these limitations. The structured report ensures completeness, accuracy, and clarity of the report and prevents inadvertently missing findings. It allows comparability and data sharing and minimizes ambiguity (12, 13). The quality of the reporting can further be improved using a standard lexicon. Several standardized tools have been described to increase the precision and conciseness of the reporting system, e.g., RECIST (Response Evaluation Criteria in Solid Tumors) and RADS (Reporting and Data System) (13). Literature is replete with examples from various subsites where the reporting quality has improved with the structured reporting. BI-RADS is one such conquest which has been widely accepted. This has led to the generation of large databases, creating the opportunity for scientists and radiologists to develop algorithms for screening and early detection (14). A study by Mc Knee et al. assessed the effect of ACR Lung-RADS in a lung screening program. The positive predictive value for diagnosed malignancy was increased from 6.9% to 17.3% in 1,603 patients with follow-up, and there was a reduction in the overall positive rate from 27.6% to 10.6% (15).

## Imaging guidelines in head and neck cancers: importance and challenges

Standardized reporting using Reporting and Data System has also been extended to head and neck cancers in the form of TI-RADS (Thyroid Imaging, Reporting and Data System) (16) and NI-RADS (Neck Imaging Reporting and Data Systems) (17). Various societies, including the American College of Radiology (ACR), have described TI-RADS. It was invented to decrease unnecessary biopsies and improve diagnostic accuracy. Interpretation and interobserver variability are common limitations of these systems (18). Attempts have been made to describe the algorithm to improve the interobserver concordance and decrease the interobserver variation. Whereas the interpretation of the individual feature may vary from operator to operator, the agreement is better based on the TI-RADS category.

There is an evolving role of deep learning/machine learning in establishing the diagnosis of these cancers. It is envisaged that artificial intelligence will save a lot of radiologists’ efforts and increase reporting efficacy. Artificial intelligence-based models, however, are challenging to report as these many times do not follow frequentist hypothesis frameworks. This also limits its broader applicability in clinical use and eternal validation. The critical challenge is to generate a generalizable model, avoiding false positive association, over and underfitting and data representation bias (19). Nevertheless, advancement in imaging is welcome in the era of precision and personalized medicine (20).

Inter-reader variability is a challenge in head and neck cancer imaging, which is more marked in the post-treatment setting. There is variability regarding the timeline and modality of the post-treatment surveillance protocol (21). Therefore, the need of the hour is to generate evidence-based guidelines and reporting lexicon to provide accurate information to the treating clinicians.

## Articles on the Research Topic

A wide variety of considerations need to be made by clinicians across all stages of the disease and throughout the treatment algorithm for numerous head and neck cancer subsites. Imaging these cancers is crucial, from initial diagnosis and treatment to monitoring treatment outcomes and detecting recurrences, metastasis, and second primaries. The Research Topic *‘Site-Specific Imaging Guidelines in Head & Neck and Skull Base Cancers’* brings together various Head and Neck site-specific cancer guidelines of the best practices as a reference point to help clinicians in decision-making and optimizing patient outcomes.

The Research Topic aims to address the evidence and data on imaging guidelines. This is the collection of 4 articles: 2 original articles, one systematic review and meta-analysis and one review article. The Research Topic comprehensively covers these topics and highlights the importance of imaging in predicting outcomes, treatment planning and managing head and neck cancers. Following are the glimpses of the articles included in this Research Topic:

Mahajan et al. analyzed the cohort of a phase III randomized controlled trial of locally advanced head and neck cancers who received concurrent chemoradiotherapy with or without nimotuzumab. NI-RADS allocation was done in 462 patients, and the study aimed to determine the accuracy of the NIRADS lexicon. Rozynek et al. developed a fully automated tissue segmentation using deep learning and investigated whether 3D body composition measurements can predict survival in head and neck cancer patients. It is an interesting article with promising results exploring deep learning in this cohort of patients. Li et al. conducted a systematic review and meta-analysis on 5,238 nodules to investigate the inter-reader agreement using ACR TI-RADS. The meta-analysis includes mainly retrospective studies and, therefore, has the limitation of selection bias and heterogeneity. Nevertheless, a well-analysed meta-analysis shows moderate inter-reader agreement between radiologists for the overall classification. Surgery and reconstruction of sinonasal cancers is a challenging issue. It is equally difficult to assess the postoperative changes and plan for adjuvant treatment due to altered anatomy and fibrosis. Carsuzaa et al. have summarized in-depth discussions of the interdisciplinary challenges and aims of reconstruction in this cohort. The article covers the reconstruction options and post-operative imaging features to help detect recurrence and plan adjuvant treatment.

To conclude, this Research Topic is an exciting issue that brings together the various articles on imaging guidelines and their impact on patient outcomes under one cover. It makes an interesting read and provides a reference point for radiologists and clinicians from multiple disciplines to manage patients more effectively.

## Author contributions

RV: Writing – original draft, Writing – review & editing. AM: Writing – original draft, Writing – review & editing. SL: Writing – original draft, Writing – review & editing. KP: Writing – original draft, Writing – review & editing. VN: Writing – original draft, Writing – review & editing. AD’C: Writing – original draft, Writing – review & editing.
